# Comparison of clinical characteristics and outcomes of pyogenic liver abscess patients < 65 years of age versus ≥ 65 years of age

**DOI:** 10.1186/s12879-019-3837-2

**Published:** 2019-03-07

**Authors:** Jia Zhang, Zhaoqing Du, Jianbin Bi, Zheng Wu, Yi Lv, Xufeng Zhang, Rongqian Wu

**Affiliations:** 1 National Local Joint Engineering Research Center for Precision Surgery & Regenerative Medicine, Shaanxi Provincial Center for Regenerative Medicine and Surgical Engineering, Institute of Advanced Surgical Technology and Engineering, First Affiliated Hospital, Xi’an Jiaotong University, 76 West Yanta Road, P.O. Box 124, Xi’an, 710061 Shaanxi Province China; 2 Department of Hepatobiliary Surgery, First Affiliated Hospital, Xi’an Jiaotong University, Xi’an, Shaanxi Province China

**Keywords:** Pyogenic liver abscess, Elderly, Comorbidities, Treatment, Prognosis

## Abstract

**Background:**

Pyogenic liver abscess (PLA) in the elderly is insufficiently elucidated. A few studies attempted to investigate the role of age in PLA have yielded controversial results. The purpose of this study was to explore the possible differences in the comorbidity, microbiological characteristics and clinical course between elderly and young PLA patients.

**Methods:**

The clinical data of 332 adult PLA patients who received treatment at our hospital from January 2010 to December 2016 were collected. The demographic data, etiologies, comorbidities, clinical features, laboratory results, imaging findings, microbiological characteristics, choices of treatment and clinical outcomes were analyzed.

**Results:**

Eighty-two (24.7%) patients were older than 65 years. Comorbidities including hypertension, diabetes mellitus, and cholelithiasis were more frequently found in older patients. Elderly PLA patients were more likely to present with atypical symptoms and signs on admission. The laboratory abnormalities and imaging findings were similar between the two groups. Klebsiella pneumonia was the most common pathogen on pus culture in both groups. There were no statistically significant differences in choices of treatment, PLA-related complications and length of in-hospital stay between the two groups. And there was no in-hospital mortality.

**Conclusions:**

The clinical characteristics were similar in young and elderly PLA patients. However, elderly PLA patients were more likely to have underlying diseases and tended to have atypical presentations. Physicians need to be vigilant when encounter possible elderly patients with PLA. However, older PLA patients had comparable outcomes as their younger counterparts. With effective treatment, both elderly and young PLA patients can be cured.

## Background

According to World Health Organization (WHO), the number of people aged 65 or older is projected to grow from an estimated 524 million in 2010 to nearly 1.5 billion in 2050. While the aging population represents a great achievement of medical advances, it also presents tremendous challenges for the public health system. Due to the progressive deterioration of the immune function with age, older people are particularly susceptible to infectious diseases. In the United States of America, elderly people (≥ 65 years of age) account for 12% of the population but almost 65% of sepsis cases [1]. Age has been shown to be an independent predictor of mortality in sepsis [1]. An epidemiology study in china also revealed that elderly sepsis patients had markedly higher mortality than their younger adult counterparts [2]. The clinical course of acute infection in elderly patients is frequently complicated by the presence of multiple chronic comorbidities. Signs and symptoms of acute infection in the elderly patients are often atypical and misleading.

Pyogenic liver abscess (PLA) is an accumulation of pus within the liver as a result of an infection. It accounts for almost half of the visceral abscess cases. Life-threatening sepsis can develop in patients with PLA. Along with the rapid aging population, both the incidence of PLA and the mean age of PLA patients have increased steadily in the past several decades [3, 4]. However, the impact of aging on PLA remains largely unknown. And there are several controversial reports on the clinical characteristics and outcomes of PLA in elderly patients [5–11]. Recent advances in antibiotic therapy, surgical techniques and intensive care have markedly improved the outcome of patients with PLA. The purpose of this study was to explore the possible differences in the comorbidity, microbiological characteristics and clinical course between elderly and young PLA patients. Here, we retrospectively analyzed the clinical data of 332 consecutive PLA patients admitted to our hospital and explored the possible differences in the comorbidity, microbiological characteristics and clinical course between elderly and young PLA patients.

## Methods

### Patients

We screened consecutive patients who were admitted to the first affiliated hospital of Xi’an Jiaotong University for treatment of PLA between January 2010 and December 2016. The diagnostic criteria were described previously [12]. This study was approved by the Ethics Committee of the First Affiliated Hospital of Xi’an Jiaotong University (XJTU1AF2015LSL-057). The patient’s informed written consent to analysis of their medical records was waived due to the retrospective nature of this study. And no further permission from the hospital was required.

### Data collection

Part of the data in this study was used to assess the impact of previous abdominal surgery on clinical characteristics and prognosis of PLA [12]. The medical records of all patients, including demographic data, etiologies, comorbidities, surgery history, clinical features, laboratory results, imaging findings, microbiological characteristics, treatments, complications and outcomes were reviewed retrospectively as we previous described [12].

### Statistical analysis

Continuous variables were presented as mean ± standard deviation (SD) and analyzed by the two-tailed Student t test. Categorical variables were presented as absolute numbers and percentages and compared by Chi-square test or Fisher exact test. Univariate and multivariate analysis of prognostic factors were performed using the logistics regression. SPSS version 22.0 (IBM, Armonk, NY) was used for statistical analysis. A two-sided *P* value < 0.05 was indicated statistical significance.

## Results

### Demographic data and comorbidities

From January 2010 to December 2016, a total of 332 adult patients were admitted to our hospital for treatment of PLA. The median age was 57 years (range 18–89). Eighty-two (24.7%) patients were older than 65 years. The demographic data, etiologies, comorbidities and surgery history were summarized in Table 1. Of the 250 young PLA patients (18–64 years of age), 59.2% were male. On the other hand, only 47.6% elderly PLA patients (≥ 65 years of age) were male (*P* = 0.065). Biliary tract disease was the most common identifiable cause of PLA in this study. More elderly PLA patients had a biliary source than their younger counterparts. On the other hand, more young PLA patients had an unknown cause than elderly PLA patients. The elderly patients were less likely to have a smoking history (15.9% vs. 30.8%, *P* = 0.008), but more likely to suffer hypertension (40.2% vs. 14.4%, *P* < 0.001), diabetes mellitus (41.5% vs. 28.8%, *P* = 0.033), cholelithiasis (50.0% vs. 32.8%, *P* = 0.005) and coronary artery disease (12.2% vs. 2.0%, *P* < 0.001) than young patients. Overall, 46.7% of the PLA patients underwent abdominal surgery before in this cohort. No difference was found in the surgery history between the two groups.

**Table 1:** Demographic data, etiologies, comorbidities and surgery history

	Total *N* = 332	Under 65 *N* = 250	Over 65 *N* = 82	*P* value
Age (years; median, range)	57(18–89)	53(18–60)	72(65–89)	
Gender (Male/Female)	187/145	148/102	39/43	0.065
Etiologies (n, %)				
Biliary source	107(32.2%)	71(28.4%)	36(43.9%)	0.009
Portal vein seeding, bowel and/or pelvic pathology	29(8.7%)	24(9.6%)	5(6.1%)	0.330
Hepatic artery seeding	19(5.7%)	16(6.4%)	3(3.7%)	0.513
Direct extension	39(11.7%)	25(10.0%)	14(17.1%)	0.084
Trauma to the liver	12(3.6%)	10(4.0%)	2(2.4%)	0.752
Cryptogenic infection	126(38.0%)	104(41.6%)	22(26.8%)	0.017
Comorbidities (n, %)				
Smoking	90(27.1%)	77(30.8%)	13(15.9%)	0.008
Drinking	56(16.9%)	46(18.4%)	10(12.2%)	0.193
Hypertension	69(20.8%)	36(14.4%)	33(40.2%)	< 0.001
Diabetes mellitus	106(31.9%)	72(28.8%)	34(41.5%)	0.033
Hepatobiliary malignant diseases	40(12.1%)	32(12.8%)	8(9.8%)	0.462
Cholelithiasis	123(37.1%)	82(32.8%)	41(50.0%)	0.005
Cirrhosis	14(4.2%)	11(4.4%)	3(3.7%)	1
Viral hepatitis	23(6.9%)	19(7.6%)	4(4.9%)	0.400
Coronary artery disease	15(4.5%)	5(2.0%)	10(12.2%)	< 0.001
Surgery history				
Abdominal surgery history	155(46.7%)	115(46.0%)	40(48.8%)	0.661
Hepatobiliary surgery	129(38.9%)	94(37.6%)	35(42.7%)	0.413
Other surgery	26(7.8%)	21(8.4%)	5(6.1%)	0.501
No surgery	177(53.3%)	135(54.0%)	42(51.2%)	0.661

### Clinical features, laboratory results and imaging findings

As shown in Table 2, fever, chills and abdominal pains were the three most common symptoms of PLA. There were no differences in these three symptoms between elderly and young PLA patients. However, more elderly PLA patients presented with nausea (*P* = 0.016) and vomit (*P* = 0.006) than young PLA patients on admission. Elderly PLA patients appeared to have a slight lower body temperature than their young counterparts (*P* = 0.062). Furthermore, elderly PLA patients had a faster heart rate than young PLA patients on admission (*P* = 0.042). In terms of laboratory results and imaging findings, however, there were no significant differences between the two groups.

**Table 2:** Clinical features, laboratory results and imaging findings

	Total *N* = 332	Under 65 *N* = 250	Over 65 *N* = 82	*P* value
Symptoms and signs (n, % or mean ± S.D.)				
Fever	292(88.0%)	221(88.4%)	71(86.6%)	0.661
Chills	170(51.2%)	131(52.4%)	39(47.6%)	0.447
Abdominal pain	144(43.4%)	105(42.0%)	39(47.6%)	0.378
Nausea	77(23.2%)	50(20.0%)	27(32.9%)	0.016
Vomit	50(15.1%)	30(12.0%)	20(24.4%)	0.006
Fatigue	55(16.6%)	44(17.6%)	11(13.4%)	0.376
Temperature (°C)	37.3 ± 1.1	37.3 ± 1.1	37.1 ± 1.0	0.062
Respiratory rate	19.8 ± 1.8	19.8 ± 1.8	19.5 ± 1.7	0.149
Heart rate	85.3 ± 13.3	86.1 ± 13.5	82.7 ± 12.5	0.042
Mean arterial pressure (mmHg)	89.8 ± 25.2	88.6 ± 25.5	93.6 ± 24.1	0.116
Laboratory results (mean ± S.D.)				
Leucocytes (× 10^9^/L)	11.1 ± 5.7	10.8 ± 5.0	12.2 ± 7.4	0.123
Neutrophils (×10^9^/L)	9.0 ± 5.5	8.7 ± 4.8	10.0 ± 7.1	0.136
Hemoglobin (g/L)	112.1 ± 19.7	112.4 ± 19.8	111.1 ± 19.7	0.624
Platelet count (× 10^9^/L)	227.6 ± 127.4	231.5 ± 133.1	215.9 ± 108.4	0.342
ALT (U/L)	64.1 ± 103.8	62.3 ± 91.3	69.8 ± 135.5	0.569
AST (U/L)	55.2 ± 139.3	50.6 ± 93.8	69.2 ± 227.7	0.295
ALP (U/L)	195.0 ± 136.2	197.2 ± 137.3	188.1 ± 133.4	0.600
GGT (U/L)	165.0 ± 158.3	159.2 ± 148.6	182.5 ± 184.3	0.248
TBIL (μmol/L)	20.7 ± 25.1	21.6 ± 27.8	18.1 ± 14.3	0.277
DBIL (μmol/L)	11.0 ± 17.4	11.7 ± 19.4	9.0 ± 8.2	0.210
ALB (g/L)	30.6 ± 5.9	30.8 ± 5.8	29.9 ± 5.9	0.200
Cr (umol/L)	65.9 ± 49.8	65.4 ± 49.8	67.1 ± 50.1	0.780
BUN (mmol/L)	5.1 ± 3.0	4.9 ± 3.1	5.6 ± 2.7	0.088
PT (s)	14.6 ± 1.8	14.5 ± 1.5	15.0 ± 2.5	0.127
APTT (s)	38.7 ± 5.7	38.6 ± 5.5	38.9 ± 6.2	0.700
INR	1.2 ± 0.2	1.2 ± 0.1	1.2 ± 0.3	0.106
FIB (g/L)	6.0 ± 1.9	6.1 ± 1.9	5.8 ± 1.8	0.198
Imaging findings (n, % or mean ± S.D.)				
Single lesion	244(73.5%)	184(73.6%)	60(73.1%)	0.939
Multiple lesions	88(26.5%)	66(26.4%)	22(26.8%)	
Maximal diameter of abscess (cm)	6.6 ± 2.8	6.6 ± 2.8	6.9 ± 2.8	0.406
Gas formation	56(16.9%)	40(16.0%)	16(19.5%)	0.461
Abscess location	*N* = 297	*N* = 229	*N* = 68	
Left lobe	45(15.2%)	34(14.9%)	11(16.2%)	0.307
Right lobe	211(71.0%)	167(72.9%)	44(64.7%)	
Both-lobes	41(13.8%)	28(12.2%)	13(19.1%)	

*ALT* Alanine Transaminase, *AST* Aspartate Transaminase, *ALP* Alkaline Phosphatase, *GGT* Gamma-Glutamyl Transpeptidase, *TBIL* Total bilirubin, *DBIL* Direct bilirubin, *ALB* Albumin, *Cr* Creatinine, *BUN* Blood Urea Nitrogen, *PT* Prothrombin Time, *APTT* Activated Partial Thromboplastin Time, *INR* International Normalized Ratio, *FIB* Fibrinogen

### Microbiological characteristics

The bacterial species identified from the patients’ samples are summarized in Table 3. Of the 332 PLA patients in this cohort, the pus culture result was available in 202 (60.8%) patients. Among them, 142 (70.3%) patients showed positive bacterial culture. Klebsiella pneumonia was the most common pathogens on pus culture in both groups. The blood culture result was available in 151 (45.5%) patients. Among them, 40 (26.5%) had an identifiable organism. Klebsiella pneumonia remained the most common pathogen in patients under 65 years of age, while Escherichia coli were the most common pathogen in patients over 65 years of age on blood culture. The elderly PLA patients appeared to have a slightly higher negative rate (no growth) on both pus and blood culture than young ones in our study. However, the differences did not reach statistically significant. Overall, no significant differences were found on the pus and blood culture results between the two groups.

**Table 3:** Microbiological characteristics

	Total	Under 65	Over 65	*P* value
Pus culture (n, %)	*N* = 202	*N* = 155	*N* = 47	
Klebsiella spp	77(38.1%)	62(40.0%)	15(31.9%)	0.317
Escherichia coli	19(9.4%)	14(9.0%)	5(10.6%)	0.777
Enterococcus	7(3.5%)	4(2.6%)	3(6.4%)	0.357
Streptococcus	8(4.0%)	8(5.2%)	0(0)	0.202
Staphylococcus	3(1.5%)	2(1.3%)	1(2.1%)	0.550
Clostridium perfringens	1(0.5%)	1(0.7%)	0(0)	1
Other	10(5.0%)	7(4.5%)	3(6.4%)	0.701
Multiple bacteria	17(8.4%)	13(8.4%)	4(8.5%)	1
No growth	60(29.7%)	44(28.4%)	16(34.0%)	0.457
Blood culture (n, %)	*N* = 151	*N* = 111	*N* = 40	
Klebsiella spp	13(8.6%)	12(10.8%)	1(2.5%)	0.186
Escherichia coli	8(5.3%)	5(4.5%)	3(7.5%)	0.437
Enterococcus	2(1.3%)	1(0.9%)	1(2.5%)	0.461
Streptococcus	4(2.7%)	3(2.7%)	1(2.5%)	1
Staphylococcus	4(2.7%)	3(2.7%)	1(2.5%)	1
Clostridium perfringens	1(0.7%)	1(0.9%)	0(0)	1
Other	3(2.0%)	3(2.7%)	0(0)	0.566
Multiple bacteria	5(3.3%)	5(4.5%)	0(0)	0.326
No growth	111(73.5%)	78(70.3%)	33(82.5%)	0.133

### Treatment and outcomes

As shown in Table 4, the majority of PLA patients in this cohort required either percutaneous or surgical drainage. Five (1.5%) patients initially treated with antibiotics alone required subsequent drainage and 2 (0.6%) patients initially treated with percutaneous drainage required surgical drainage. There were 44 PLA patients with gallstones in this study. Twenty patients had a cholecystectomy at the time of abscess drainage. Others were managed with antibiotics alone (*n* = 8), percutaneous drainage (*n* = 11) and surgical drainage (*n* = 5). In young PLA patients, 26.0% were managed with antibiotics alone, 59.2% required percutaneous drainage, and 14.8% required surgical drainage. In elderly PLA patients, on the other hand, 37.8% were managed with antibiotics alone, 48.8% required percutaneous drainage, and 13.4% required surgical drainage. A total of 170 patients (51.2%) received empirical antibiotic treatments in this study. There were no statistically significant differences in the percentage of patients received empirical antibiotic treatments between the two groups. The proportion of patients who required percutaneous or surgical drainage was also similar between the two groups (*P* = 0.120, Table 4). There were no statistically significant differences in length of antibiotics required between young and older PLA patients. Interestingly, days taken for temperature normalization were significantly shorter in elderly PLA patients than young ones (*P* = 0.040, Table 4). However, there were no differences in the incidence of PLA-related complications and length of in-hospital stay between the two groups. The number of patients received antibiotic therapy in the preceding 3 months and required re-operation were also similar between young and elderly groups (Table 4). Only 16 patients required ICU care in this study. There was no significant difference in the length of ICU stay between the groups. And there was no in-hospital mortality in this cohort (Table 4).

**Table 4:** Treatments, complications and outcomes

	Total *N* = 332	Under 65 *N* = 250	Over 65 *N* = 82	*P* value
Treatments (n, %)				
Empirical antibiotic treatment	170(51.2%)	135(54.0%)	35(42.7%)	0.075
Antibiotics alone	96(28.9%)	65(26.0%)	31(37.8%)	0.120
Percutaneous drainage	188(56.6%)	148(59.2%)	40(48.8%)	
Surgical drainage	48(14.5%)	37(14.8%)	11(13.4%)	
Complications (n, %)				
Sepsis	151(45.5%)	111(44.4%)	40(48.8%)	0.489
Septic shock	3(0.9%)	2(0.8%)	1(1.2%)	0.574
Acute Respiratory Distress Syndrome	3(0.9%)	3(1.2%)	0(0)	1
Acute kidney injury	1(0.3%)	1(0.4%)	0(0)	1
Spontaneous rupture of abscess	2(0.6%)	1(0.4%)	1(1.2%)	0.434
Pleural effusion	117(35.2%)	87(34.8%)	30(36.6%)	0.769
Portal venous thrombosis	2(0.6%)	2(0.8%)	0(0)	1
Metastatic complications	8(2.4%)	7(2.8%)	1(1.2%)	0.693
Outcomes (% or mean ± S.D.)				
Length of antibiotics required (days)	8.4 ± 5.3	8.3 ± 5.4	8.7 ± 4.9	0.535
Time taken for temperature normalization (days)	7.0 ± 6.1	7.4 ± 6.3	5.8 ± 5.3	0.040
Length of hospital stay (days)	15.6 ± 8.3	15.9 ± 8.3	14.7 ± 8.4	0.258
Received antibiotic therapy in the preceding 3 months	62(18.7%)	43(17.2%)	19(23.2%)	0.229
Re-operated	12(3.6%)	12(4.8%)	0(0)	0.093
In-hospital mortality	0	0	0	

### Prognostic factors associated with the development of sepsis in PLA patients

Sepsis is a common and serious complication of PLA. In this study, a total of 154 patients (46.4%) developed sepsis or septic shock. As shown in Table 5, the development of sepsis or septic shock was significantly associated with hepatic artery seeding, cryptogenic infection, history of alcohol drinking and previous abdominal surgery in the univariate analysis. In the multivariate analysis, however, only hepatic artery seeding remained independently associated with the development of sepsis.

**Table 5:** Prognostic factors associated with the development of sepsis and septic shock in PLA patients

Variable (*N* = 332)	Univariate analysis			Multivariate analysis	
	Yes *N* = 154	No *N* = 178	*P* value	OR (95% CI)	*P* value
Age (years; median, range)	56(18–85)	59(20–89)	0.290		
Gender (Male/Female)	88/66	99/79	0.780		
Etiologies (n, %)					
Biliary source	53(34.1%)	54(30.3%)	0.428		
Portal vein seeding, bowel and/or pelvic pathology	17(11.0%)	12(6.7%)	0.167		
Hepatic artery seeding	17(11.0%)	2(1.1%)	< 0.001	0.105(0.023–0.486)	0.004
Direct extension	17(11.0%)	22(12.4%)	0.709		
Trauma to the liver	5(3.2%)	7(3.9%)	0.738		
Cryptogenic infection	45(29.2%)	81(45.5%)	0.002	1.406(0.824–2.397)	0.211
Comorbidities (n, %)					
Smoking	49(31.8%)	41(23.0%)	0.073		
Drinking	33(21.4%)	23(12.9%)	0.039	0.617(0.329–1.154)	0.131
Hypertension	28(18.2%)	41(23.0%)	0.277		
Diabetes mellitus	56(36.4%)	50(28.1%)	0.107		
Hepatobiliary malignant diseases	20(13.0%)	20(11.2%)	0.625		
Cholelithiasis	58(37.7%)	65(36.5%)	0.829		
Cirrhosis	4(2.6%)	10(5.6%)	0.170		
Viral hepatitis	8(5.2%)	15(8.4%)	0.886		
Coronary artery disease	5(3.2%)	10(5.6%)	0.413		
Surgery history					
Abdominal surgery history	81(52.6%)	74(41.6%)	0.045	0.617(0.368–1.035)	0.067
Hepatobiliary surgery	67(43.5%)	62(34.8%)	0.106		
Other surgery	14(9.1%)	12(6.7%)	0.427		

### Prognostic factors associated with prolonged time (≥7 days) taken for temperature normalization in PLA patients

Normalization of body temperature is an indicator of recovery in PLA patients. A multivariate analysis was performed to determine the independent factors associated with prolonged time (≥7 days) taken for temperature normalization in PLA patients. As shown in Table 6, male and alcohol drinking were associated with shorter time taken for temperature normalization in PLA patients.

**Table 6:** Prognostic factors associated with prolonged time (≥7 days) taken for temperature normalization in PLA patients

Variable (*N* = 332)	Univariate analysis			Multivariate analysis	
	< 7 days *N* = 174	≥ 7 days *N* = 158	*P* value	OR (95% CI)	*P* value
Age (years; median, range)	57(20–89)	59(18–84)	0.385		
Gender (Male/Female)	108/66	79/79	0.027	1.767(1.017–3.070)	0.012
Etiologies (n, %)					
Biliary source	64(36.8%)	43(27.2%)	0.063		
Portal vein seeding, bowel and/or pelvic pathology	13(7.5%)	16(10.1%)	0.392		
Hepatic artery seeding	6(3.4%)	12(7.6%)	0.162		
Direct extension	18(10.3%)	21(13.3%)	0.405		
Trauma to the liver	7(4.0%)	5(3.2%)	0.676		
Cryptogenic infection	65(37.4%)	61(38.6%)	0.814		
Comorbidities (n, %)					
Smoking	52(29.9%)	38(24.1%)	0.232		
Drinking	38(21.8%)	18(11.4%)	0.011	2.849(1.262–6.430)	0.012
Hypertension	40(23.0%)	29(18.4%)	0.299		
Diabetes mellitus	58(33.3%)	48(30.4%)	0.564		
Hepatobiliary malignant diseases	19(10.9%)	21(13.3%)	0.507		
Cholelithiasis	74(42.5%)	49(31.0%)	0.052		
Cirrhosis	10(5.7%)	4(2.5%)	0.145		
Viral hepatitis	14(8.0%)	9(5.7%)	0.400		
Surgery history					
Abdominal surgery history	87(50.0%)	68(43.0%)	0.204		
Hepatobiliary surgery	74(42.5%)	55(34.8%)	0.150		
Other surgery	13(8.5%)	13(8.2%)	0.798		

## Discussion

Clinical characteristics and outcomes of PLA in elderly patients are insufficiently elucidated. A few studies attempted to investigate the role of age in PLA have yielded controversial results [5–10]. In the current study, we found that elderly PLA patients were more likely to have underlying diseases and present with atypical symptoms and signs on admission. However, the microbiological characteristics and clinical courses of young and elderly PLA patients were similar. More importantly, there were no major differences in the overall outcomes between young and elderly PLA patients.

Comorbidities such as hypertension, diabetes mellitus, and cholelithiasis were more frequently found in older patients. This is expected as it reflects a greater prevalence of these diseases in the elderly population. In the current study, we also found that men under 65 were more likely to develop PLA than women; however, the PLA incidence appeared to increase in elderly women. This result is consistent with several previous observations [5, 6]. Hormonally active women are better protected from sepsis than men [13, 14]. This gender bias may be attributed to female sex hormones. Sex hormones play an important role in inflammatory responses [14–18]. Animal studies have consistently shown a survival advantage in females in critical illness including sepsis [19–21]. Estrogen administration or blockade of the testosterone receptor has been shown to reduce organ injury in experimental models of sepsis [13, 22, 23]. Thus, the trend in gender distribution with age can be explained by the reduced estrogen level in postmenopausal women which makes them more susceptible to PLA than their younger counterparts.

The clinical presentations, laboratory abnormalities, imaging findings and microbiological characteristics were similar in the two groups. However, the elderly patients had a lower body temperature and a higher heart rate than young patients in our study. In addition, the elderly PLA patients were more likely to have non-specific gastrointestinal complaints such as nausea and vomit than their younger counterparts on admission. Consistent with findings in other PLA studies conducted in Asia [24–27], the most frequent pathogen identified in this study was Klebsiella pneumonia. However, the elderly PLA patients appeared to have a slightly lower positive rate on both pus and blood culture than young ones in our study. Thus, the diagnosis of PLA can be challenging in the geriatric population. Clinicians need to be vigilant when encounter elderly patients with atypical symptoms and signs of PLA.

In this study, the patients were treated by physician discretion based on each patient’s condition. In general, selection of therapeutic methods was dependent on the number and size of abscesses, degree of abscess liquefaction, separation of abscess cavity, with/without other comorbidities, patients’ response to antibiotics and personal experience of the physicians. For the method of drainage, percutaneous treatment was first taken into consideration. However, surgical drainage was used if the diameter of the abscess was larger than 5 cm, multilocular abscesses were present, percutaneous drainage failed, or when surgical treatment of the underlying cause of PLA was required [28].

Advanced age is an important contributor to morbidity and mortality in patients with sepsis [1]. However, the impact of aging on outcomes of patients with PLA remains unclear. Some studies have indicated that older age was associated with increased mortality in PLA [6, 29], while others have shown that older PLA patients had a fair or similar outcome compared with their younger counterparts [5, 7]. In terms of the treatment options, the majority of PLA patients in this cohort required either percutaneous or surgical drainage. We did not find any significant differences in the therapeutic procedures performed between young and elderly PLA patients. More importantly, elderly and young PLA patients had a similar clinical outcome in the current study. We did not find any significant differences in PLA-related complications between young and elderly PLA patients. And it even took less time for elderly PLA patients’ temperature to return to normal than young ones. However, this does not necessary mean elderly PLA patients recover faster than young patients, as elderly PLA patients had slight lower body temperatures than young ones on admission. Owing to advances in imaging techniques and novel antibiotics, mortality from PLA has been steadily decreasing during the past several decades [3, 4]. In this cohort, no patients died during their stay in the hospital. This result demonstrates that with effective treatment both elderly and young PLA patients can be cured.

Several limitations of this study need to be considered. First, we only included patients from a single center. Substantial differences in etiology, treatment and outcomes of PLA have been revealed in studies from different regions [30]. Therefore, our findings need to be validated by multicenter studies. Second, we only investigated the short-term outcomes of PLA in this study. This is due to the consideration that the underlying disease would significantly influence the long-term outcomes of the patient. And life-expectancy is expected to be shorter in elderly patients. To evaluate the impact of aging on the long-term outcomes of PLA, a prospective propensity score-matched study is warranted in the future. Finally, this is a retrospective study. The results are subject to a selection bias, recall bias and some residual confounding. A prospective multicentric study should be performed to validate our findings.

## Conclusions

The clinical presentations, laboratory abnormalities, imaging findings and microbiological characteristics were similar in young and elderly PLA patients. However, elderly PLA patients were more likely to have underlying diseases and tended to present with atypical symptoms and signs on admission. Physicians need to be on high alert when encounter possible elderly PLA patients. However, older PLA patients had comparable outcomes as their younger counterparts. With effective treatment, both elderly and young PLA patients can be cured.

## Abbreviations

ALP: Alkaline phosphatase
ALT: Alanine aminotransferase
APTT: Activated partial thromboplastin time
AST: Aspartate transaminase
BUN: Blood urea nitrogen
Cr: Creatinine
CT: Computed tomography
DBIL: Direct bilirubin
FIB: Fibrinogen
GGT: Gamma-glutamyl transferase
INR: International normalized ratio
PLA: Pyogenic liver abscess
PT: Prothrombin time
SD: Standard deviation
TBIL: Total bilirubin
